# Advances and Challenges in Understanding Cerebral Toxoplasmosis

**DOI:** 10.3389/fimmu.2019.00242

**Published:** 2019-02-14

**Authors:** Dirk Schlüter, Antonio Barragan

**Affiliations:** ^1^Hannover Medical School, Institute of Medical Microbiology and Hospital Epidemiology, Hannover, Germany; ^2^Department of Molecular Biosciences, The Wenner-Gren Institute, Stockholm University, Stockholm, Sweden

**Keywords:** MeSH, neurotropic agents, neuroparasite, neuroinflammation, CNS infection, congenital infection

## Abstract

*Toxoplasma gondii* is a widespread parasitic pathogen that infects over one third of the global human population. The parasite invades and chronically persists in the central nervous system (CNS) of the infected host. Parasite spread and persistence is intimately linked to an ensuing immune response, which does not only limit parasite-induced damage but also may facilitate dissemination and induce parasite-associated immunopathology. Here, we discuss various aspects of toxoplasmosis where knowledge is scarce or controversial and, the recent advances in the understanding of the delicate interplay of *T. gondii* with the immune system in experimental and clinical settings. This includes mechanisms for parasite passage from the circulation into the brain parenchyma across the blood-brain barrier during primary acute infection. Later, as chronic latent infection sets in with control of the parasite in the brain parenchyma, the roles of the inflammatory response and of immune cell responses in this phase of the disease are discussed. Additionally, the function of brain resident cell populations is delineated, i.e., how neurons, astrocytes and microglia serve both as target cells for the parasite but also actively contribute to the immune response. As the infection can reactivate in the CNS of immune-compromised individuals, we bring up the immunopathogenesis of reactivated toxoplasmosis, including the special case of congenital CNS manifestations. The relevance, advantages and limitations of rodent infection models for the understanding of human cerebral toxoplasmosis are discussed. Finally, this review pinpoints questions that may represent challenges to experimental and clinical science with respect to improved diagnostics, pharmacological treatments and immunotherapies.

## Introduction

In 1908, Nicolle and Manceaux discovered a bow (Greek: *toxon*) -shaped (Greek: *plasma*) parasite in the gundi (Ctenodactylus gundi), a North African rodent. Independently, Splendore (1908) described the parasite in a rabbit. The definitive hosts of *Toxoplasma gondii* are Felidae, e.g., domestic cats and other felines in which this obligate intracellular apicomplexan parasite replicates sexually in their intestine. Humans and many other warm-blooded vertebrates, e.g., rodents and birds, serve as intermediate hosts for the parasite and may become infected by oral ingestion of oocysts shed by felines or by tissue cysts that persist in the muscles and nervous system of the intermediate hosts (1).

Population structure studies of *T. gondii* have revealed that a few major clonal lineages predominate in different global geographical regions (2). Humans in Western Europe and North America are mainly infected by type II strains of *T. gondii* but type I, type III and so-called atypical strains can also infect humans and may cause disease (3). In the human host, orally ingested parasites are released from oocysts or tissue cysts (containing bradyzoite stages) and invade intestinal epithelial cells to transform into fast replicating tachyzoite stages. These tachyzoites multiply intracellularly in a parasitophorous vacuole (PV), which separates the parasite from the host cell cytoplasm. Unrestricted parasite replication results finally in the rupture of the host cell, release of tachyzoites and infection of neighboring cells. Due to the ensuing immune response and the production of anti-parasitic intracellular effector molecules by the infected cells, the tachyzoites may be either eliminated or transform into slow replicating bradyzoites, which form intracellular persisting cysts. These cysts may contain hundreds of parasites and preferentially form in neurons of the brain and retina as well as in muscle cells. It is thought that also at this chronic stage of infection, the immune system plays a critical role in parasite control but may also contribute to disease, particularly in the eye.

In healthy humans, most primary infections with *T. gondii* are asymptomatic or cause only mild symptoms including malaise, swelling of lymph nodes and fever (4). However, primary infection during pregnancy may be harmful or even fatal to the fetus. Bradyzoite-containing brain cysts may become reactivated, i.e., reconvert into cytotoxic tachyzoites during secondary immune deficiency and cause toxoplasmic encephalitis (TE), in particular in individuals with HIV/AIDS or organ transplants. Ocular toxoplasmosis (OT) was identified decades ago as a manifestation of congenital toxoplasmosis but in the last years it has been uncovered that OT may also develop upon primary infection of immune-competent adults and has a high risk for recrudescence even upon anti-parasitic treatment.

Here, we discuss recent advances in the understanding of the delicate interplay between *T. gondii*, infected cells and the immune system in experimental and clinical settings. The major focus is on parasite invasion into the brain, mechanisms of parasite control in the brain and factors leading to reactivation of chronic cerebral toxoplasmosis. In particular, we delineate similarities, differences and open questions between rodent models, and human cerebral toxoplasmosis. We briefly address some aspects on the impact of the parasite on the brain neurophysiology. However, for this specific aspect, we refer the reader primarily to recent reviews (5–8).

## Establishment of Infection in the CNS

The mechanisms that lie behind the systemic dissemination and the parasite's penetration to the human brain are largely unknown. The concepts are thus mainly based on an extrapolation from studies in rodents, which also are natural hosts for Toxoplasma (Table 1).

**Table 1:** Clinical manifestations of toxoplasmosis in humans and available mouse models, with special reference to disease in the CNS and immunoprivileged organs.

**Organ/tissue compartment or condition**	**Parasite stage**	**Clinical manifestation or condition in humans**	**References**	**Experimental mouse model or *in vitro* model**	**References**
Intestine	Oocyst/bradyzoite	Considered asymptomatic. Colitis in immunocompromised patients (rare)	(4) (9)	*In vivo* intestinal dissemination and inflammation models	(10–12)
Blood	Tachyzoite	Parasitemia in disseminated toxoplasmosis	(13)	*In vivo* dissemination models	(14, 15)
Blood-brain barrier/brain vasculature	Tachyzoite	Meningo-encephalitis	(4, 16)	*In vitro* and *in vivo* models	(17, 18)
Meninges/plexus choroideus	Tachy/bradyzoite	Meningo-encephalitis	(19, 20)	*In vivo* models	(21, 22)
Brain parenchyma	Tachy/bradyzoite	Encephalitis	(23)	*In vivo* model	(24)
Brain parenchyma	Bradyzoite	Chronic latent infection	(25)	*In vivo* model	(26)
Spinal cord	Tachy/bradyzoite	Myelitis (rare)	(27)	*In vivo* model	(28)
Eye	Tachy/bradyzoite	Retinochoroiditis	(29)	*Ex vivo* and *in vivo* models	(30, 31)
Testis	Tachyzoite	Orchitis (rare)	(32)	Acute testis infection	(33)
Lungs	Tachyzoite	Pneumonia	(34, 35)	Lung infection	(36)
Placenta	Tachyzoite	Congenital neurotoxoplasmosis	(37, 38)	*In vitro* and *in vivo* models	(39, 40)
Systemic immunosupressive conditions	Tachy/bradyzoite	Reactivation during HIV/AIDS Organ transplantation/chemotherapy	(41) (42)	Dexamethasone/IFN-γ R^−/−^ mice combined with bioluminescence	(21, 22)
CNS	Tachy/bradyzoite	Immune reconstitution inflammatory syndrome	(43)	Not defined	

### Predilection of the Infection for the CNS

Although Toxoplasma undoubtedly causes pathology in the CNS and is often presented as a neurotropic parasite, there is some debate regarding its neurotropism, that is, whether its affinity for the CNS is superior or different to that for other organs. During acute infection, *T. gondii* disseminates broadly but parasite loads in mice are, in fact, substantially lower in the CNS compared with other organs. Thus, Toxoplasma can hardly be classified as a primarily neurotropic pathogen having a selective higher affinity for the CNS over other organs. However, seemingly common for human and rodent infections, the parasite overcomes the restrictiveness of the blood-brain barrier (BBB) to establish infection. In fact, *T. gondii* can infect virtually any nucleated cell within and outside the CNS. Yet, as chronic infection sets in, parasite cysts are found in the CNS (and musculature) and persist in the CNS while they are cleared from peripheral organs over time. In that sense, infection can be considered neurotropic because chronic reactivated infection and congenital infection manifest primarily in the CNS. Although possible associations with parasite virulence determinants such as ROP18 have recently been put forward (44), molecular evidence is missing of parasite-derived mechanisms that would define tropism for the CNS over other organs. Various aspects of the immunopathogenesis linked to the persistence of the parasite in the CNS are discussed below.

### Systemic Dissemination: How do Toxoplasma Tachyzoites Reach the Brain?

Following oral ingestion of tissue cysts (45) or oocysts (46), the parasites invade the intestinal tissue and rapidly differentiate into tachyzoites. In experimental oral or intra-peritoneal infections in mice, parasites are rapidly found in the mesenteric lymph nodes and in the blood circulation, indicating that a lymphatic-to-blood route is likely. It remains however unknown if a direct hematogenic route, i.e., from the gut tissue directly into the blood can be taken for systemic dissemination, as suggested for Salmonella (47).

How is Toxoplasma transported in the blood circulation to ultimately reach the BBB? In experimental murine models, leukocyte-associated parasites are rapidly detected in the blood while extracellular/free parasites in the blood are also present later during infection (18, 48). It remains unclear if free tachyzoites in the blood are part of the natural infection or a consequence of high parasitic loads in experimental settings. In natural infections in rodents, invasion of the CNS is accomplished with relatively low parasitemia. Regardless, given the sensitivity of free tachyzoites to neutralization by the complement system and IgM (49), leukocytes offer a safe intracellular niche for hematogenic dissemination. Also, leukocytes have been reported to deliver parasites after egress in the lung tissue (36). Different types of leukocytes can be infected and parasite transfer between leukocyte types occurs in the blood compartment and in peripheral tissues (15, 50). In this context, Toxoplasma induces a hypermigratory phenotype in human and mouse dendritic cells (DCs) (51, 52). Hypermigration of infected DCs potentiates systemic parasite dissemination in mice, including the CNS, and may cooperate with chemotactic responses of DCs (53, 54). However, hypermigration has not been linked specifically to passage across the BBB *in vivo* as discussed below (15).

It remains unknown if the recently discovered lymphatic system of the meningeal vessels (*dura mater*) in the brain (55, 56) plays a role for the systemic dissemination into the CNS or out from the CNS upon reactivated toxoplasmosis. However, the absence of dominant meningeal manifestations during cerebral toxoplasmosis advocates against a detrimental role. Similarly, no evidence indicates that the fenestrated blood vessels of the choroid plexus would be a preferred or predominant locus for CNS invasion, with penetration to the cerebrospinal fluid (CSF) and further spread to the parenchyma (22). In clinical settings, Toxoplasma is infrequently detected in the CSF by PCR and, thus, not recommended as a standard diagnostic procedure in AIDS patients with suspected reactivated TE. It is therefore likely that passage of Toxoplasma from the blood circulation is effectuated mainly across the parenchymal vasculature.

### Entry Into the CNS by Breaching the Blood-Brain Barrier (BBB)

In order to reach the brain parenchyma from the cerebral blood circulation, Toxoplasma has to relate to the brain endothelium, primarily to the capillary bedding. There is *in vitro* evidence that Toxoplasma-infected leukocytes and free tachyzoites can transmigrate across epithelial/endothelial cell monolayers (17, 51, 57, 58) and likely extravasate *in vivo* in mice (59). More recent studies have put forward that infection of endothelial cells occurs and is necessary for penetration to the CNS (18). One additional possibility is that leukocytes “deliver” the parasites to the endothelium (60), as recently suggested in a pulmonary infection model in mice (36). It remains unknown how Toxoplasma crosses the BBB below the endothelial cell linings, i.e., basal lamina and astrocytic end-feet. Similarly, the eye is an immune-privileged site and similar mechanisms have been put forward for passage of the blood-retina barrier, implicating both Trojan horse mechanisms and free extracellular tachyzoites (30, 61, 62).

Where and when does Toxoplasma cross the vasculature of the BBB? While Plasmodium, causing cerebral malaria, seems to have a predilection for adhesion to post-capillary venules (63) with a possible involvement of leukocytes (64), it remains unknown if Toxoplasma crosses at the level of arterioles, capillaries or post-capillary venules. There is no evidence of a hemorrhagic process upon penetration to the CNS. However, are there perturbations in the permeability of the BBB, albeit locally, upon Toxoplasma invasion to the parenchyma? Recent work suggests that vascular dysfunction persists during chronic infection (65). The inflammatory or anti-inflammatory components that may accompany or facilitate passage across the BBB during acute infection also remain enigmatic. Future research needs to address these questions in order to understand passage of Toxoplasma to the brain parenchyma.

The kinetics of passage to the parenchyma also remains poorly understood. Early histological studies detected tachyzoites in the brain parenchyma by day 5 after intravenous inoculation of mice (66). Detection of parasites in cerebral homogenate by bioassay/plaquing assays or qPCR was earliest on day 6–7 post-infection in oral experimental infections (45, 59) and by day 3–4 after intraperitoneal inoculation in blood-perfused mice (15).

Jointly, mounting evidence show that Toxoplasma utilizes combined strategies for systemic dissemination (60), by hijacking leukocytes (51, 59, 67) and, as free parasites (18, 48), with significant differences between Toxoplasma genotypes (48, 68). If these combined strategies are also utilized to enforce the BBB awaits future investigations. Also, Toxoplasma-derived factors directly implicated in cerebral toxoplasmosis remain to date unidentified.

## Chronic Infection in the Brain Parenchyma

The mechanisms leading to parasite persistence and control in the human brain are largely unknown. The concepts are therefore mainly based on extrapolations from experimental studies in rodents (Table 1).

### Parasite Localization and Cell Preference in the CNS

Primary Toxoplasma infection in humans is, in general, clinically asymptomatic or accompanied by discrete symptomatology (4). Yet, based on murine models, the parasite penetrates to the brain parenchyma during this initial phase (59). At the BBB and after contact with the endothelium, the parasite must cross one additional layer of the barrier that is constituted by the basal lamina and the astrocytic end-feet. Yet, little evidence of astrocytic infection was found during this process (26), indirectly supporting the concept that carrier immune cells could mediate transportation (26, 59). One alternative possibility is that microglia that also express a hypermigratory phenotype upon Toxoplasma infection serve as “Trojan horses” for parasitic spread in the parenchyma (69).

Studies in mice have shown that Toxoplasma cysts can be retrieved from any anatomical brain area (70) and that cyst abundance can be higher in the amygdala, thalamus and striatum (71, 72). This anatomic distribution of cysts is likely an important factor contributing to the manipulation of murine behavior by the parasite (70). Murine reactivation/recrudescence models provided additional information and illustrated that reactivation occurred preferentially in the gray matter of the frontal and parietal cortex, compared to cerebellum or other cortical areas (22). This distribution pattern of cortical parasitic foci may be consistent with blood transportation by the middle cerebral artery and extravasation from its ramifications during primary infection.

Although astrocytes, microglia and neurons can readily be infected *in vitro* (73), a preference for parasite localization to neurons upon cyst formation has originally been identified by Ferguson and Hutchison (74) and confirmed by others (26, 72). Exactly how *Toxoplasma* reaches neurons is not understood and the mechanisms allowing parasite persistence in neurons, and not astrocytes remain enigmatic (75). Neurons express MHC class I under certain circumstances, including activity-dependent, long-term structural and synaptic modifications (76, 77), and in functionally inactive IFN-γ-stimulated neurons (78). However, it is at present an open question whether all neurons infected with Toxoplasma tachyzoites or cysts express MHC class I and present parasitic antigens to CD8^+^ T cells. Eventually, some cyst-harboring neurons remain MHC class I negative and, thus, escape elimination by CD8^+^ T cells, which in principle effectively remove cysts from the brain in a perforin-dependent manner (79). Additionally, genetic fate mapping studies have demonstrated that *T. gondii* tachyzoites infect neurons but not astrocytes in mice and, thus, the absence of infected astrocytes may not be caused by an elimination of tachyzoites by astrocytes *in vivo* (26).

It has been suggested that *T. gondii* tachyzoite to bradyzoite conversion and cyst formation primarily occurs in post-mitotic cells such as neurons and muscle cells (80), which may even induce spontaneous bradyzoite formation without stimulation by the immune system (81). However, in the retina, both neurons and glial cells, which have the capacity to proliferate, harbor Toxoplasma cysts (82). Additionally, a Toxoplasma line was reported to convert spontaneously from tachyzoites to cyst-forming bradyzoites in human cell lines including epithelial cells, fibroblasts, muscle cell, and glial cells (83). Collectively, these data indicate that depending on the parasite strain both proliferating and post-mitotic host cells allow spontaneous cyst formation *in vivo* and *in vitro*.

### Role of the Host and Parasite Genetic Backgrounds for Persistence in the CNS

Without doubt, the infection has a clear predilection for the CNS, which plays an important role for the clinical manifestations of human toxoplasmosis. Even in congenital toxoplasmosis, when the parasite could disseminate to all organs of the highly immune-compromised fetus, the preferentially infected organs are the brain and the eyes. Based on data from rodent infection models, it is generally assumed that, following primary infection, *T. gondii* establishes a life-lasting chronic infection in the brain. But, does *T. gondii* always persist after primary infection in humans? This notion is based on clinical and experimental data:

- *T. gondii* seropositive AIDS patients, with low CD4^+^ T cell count (<200/μl) and with specific antibodies against *T. gondii* proving prior infection, are at risk of developing a reactivated TE due to the loss of T cell-mediated control of persisting brain cysts (23).
- *T. gondii* seropositive patients receiving immunosuppressive therapies, e.g., upon hematopoietic stem cell transplantation (HSCT) and solid organ transplantation (SOT), are at risk of developing a reactivated toxoplasmosis (23).
- In very few cases, intracerebral *T. gondii* cysts have been detected by immunohistochemistry in brain autopsies of immunocompetent patients, who died from unrelated diseases (25).
- Experimental studies, predominantly in mice but also in other species, have shown that upon experimental infection, *T. gondii* cysts persist in the CNS (84).

However, it remains questionable whether *T. gondii* persists for a lifetime in all infected humans. In fact, only ≈30% of *T. gondii* seropositive AIDS patients will develop a reactivated TE, even if the CD4^+^ T cell count is very low (23). In addition, only a very reduced number of *T. gondii* seropositive patients undergoing immunosuppression due to HSCT, develop a reactivated systemic or cerebral toxoplasmosis (85). In *T. gondii* seropositive AIDS and HSCT patients without reactivated TE, it is unclear whether other immune mechanism(s) compensate for the T cell deficiency and control the parasite or whether the parasite failed to persist in the brain. Additionally, the number of autopsy cases identifying asymptomatically persisting *T. gondii* cysts in the brain is surprisingly low. It might be argued that pathologists ought to accidentally detect *T. gondii* cysts more frequently based on the high seropositivity rates of adults (ranging from 20 to 80% depending on the age and geographical region), though it might be difficult to detect some few cysts in the large brain volume.

Parasite virulence and persistence in mice is strongly dependent on the host genetic background (86). Certain mouse strains harbor a high intracerebral parasite load upon experimental infection and might—in contrast to humans—even succumb due to the overwhelming infection. In contrast, other mouse strains have lower cyst counts in the brain and in certain rat strains the parasite even fails to persist in the CNS. Detailed studies in mice have linked relative resistance to the infection, i.e., time of survival and intracerebral parasite numbers, to genes inside and outside the MHC locus (87). Outbred mice (e.g., NMRI) and mouse strains with the H-2^d^ MHC haplotype (e.g., BALB/c and B6.C H-2^d^ mice) develop a chronic latent cerebral toxoplasmosis and survive the infection with low cyst numbers and low inflammation, whereas mice with the H-2^b^ (e.g., C57BL/6 mice) and H-2K background (e.g., CBA/Ca mice) develop a lethal chronic progressive TE with high parasite loads and extensive inflammation (88, 89). Additionally, the genotype of the parasite might also have an impact on cyst formation. Most parasite genotypes, such as type II strains that commonly infect humans, form cysts in mice and humans. In contrast, prototypic type I strains rapidly cause lethal infection in mice before cyst formation but appear to form cysts and generate chronic infection in other species, including humans (90–92). However, even among type II strains, a range of virulence is observed in mice. Finally, the number of ingested parasites impacts on the outcome of infections and severity of cerebral toxoplasmosis, adding another layer of complexity. The relative importance of host- and parasite-specific factors but also of experimental conditions on the course of murine toxoplasmosis has recently been discussed (93).

In conclusion, parasite persistence varies significantly between host species and depends on the genetic backgrounds of both host and parasite. Altogether, it is reasonable to speculate that prolonged or life-long parasite persistence in the human brain may occur only in a fraction of *T. gondii* seropositive individuals. Due to the difficulties in determining and quantifying parasite persistence in the human brain, the extent of cyst formation remains unresolved in humans. Here, PCR analysis of larger brain tissue samples from *T. gondii* positive autopsy cases might clarify the ratio of parasite persistence *vs*. seropositivity.

### Immune-Mediated Control of *T. gondii* and Immunopathology in the CNS

Cell-mediated immunity to *T. gondii* includes activation of innate immune cells as well as antigen-specific T and B cells. In mice, infection with *T. gondii* rapidly induces the MyD88-dependent production of IL-12 by DCs. In particular, *T. gondii* profilin activates TLR11 and TLR12 in CD8α^+^ DCs and also TLR12 in plasmacytoid DCs. Although humans lack TLR11 and TLR12, other TLRs may induce IL-12 production upon engagement with *T. gondii*, since parasite RNA and DNA is also recognized by TLR3, 7, and 9. In the early phase of toxoplasmosis, the IFN-γ production by NK cells and type I innate lymphoid cells is important for parasite control (94). Beside IL-12, presentation of parasitic antigens by DCs and macrophages is required for activation and expansion of parasite-specific CD4^+^ and CD8^+^ T cells. CD4^+^ and CD8^+^ T cells also produce IFN-γ establishing another layer of protection, which can also compensate for insufficient IFN-γ production by NK cells (95).

In parallel to the parasite spread to the brain, inflammatory leukocytes are recruited to the CNS. These inflammatory infiltrates are mainly composed of CD4^+^ and CD8^+^ T cells with the addition of F4/80^+^ macrophages, CD11c^+^ DCs, and Ly6C^high^ inflammatory monocytes. CD8^+^ T cells recognize secreted *T. gondii* antigens (96) and play a key role in the control of cerebral toxoplasmosis, illustrated by the fact that resistance is linked to the L^d^ MHC class I gene. The parasitic GRA6 antigen is processed to a decamer epitope presented by L^d^, eliciting a robust and immunodominant CD8^+^ T cells response (97). In contrast, an immunodominant CD8^+^ T cell epitope is missing in H-2^b^ mice, which might explain why CD4^+^ and CD8^+^ T cells are both important for control of *T. gondii* in H-2^b^ mice (98), whereas CD8^+^ T cells play a more important role in H-2^d^ mice (99).

The major protective mechanisms of CD4^+^ and CD8^+^ T cells in cerebral toxoplasmosis are the production of protective cytokines, in particular IFN-γ and TNF. Perforin-dependent cytotoxicity seems to play some role for the control of *T. gondii* in the CNS but this protective pathway might be of limited importance since the majority of infected cells are MHC class I^−^ neurons. In support, intravital microscopy studies revealed that parasite-specific CD8^+^ T cells transiently interact with CD11b^+^ cells, which might be microglia, macrophages, monocytes and DCs presenting *T. gondii* antigen, but not with *T. gondii*-infected neurons (100, 101).

In addition to T cells, several types of myeloid cells, including CD8α^+^CD11c^+^ lymphoid DCs, CD11b^+^CD11c^+^ myeloid DCs and PDCA^+^B220^+^ plasmacytoid DCs, are recruited to the *T. gondii*-infected brain. Also, in the CNS, DCs produce IL-12 that may contribute to the maintenance of IFN-γ production of T cells (102). In addition, inflammatory Ly6C^hi^CCR2^+^GR1^+^ monocytes contribute to parasite control by the production of protective cytokines (IL-1α, IL-1β, IL-6, TNF) and anti-parasitic effector molecules (reactive oxygen species (ROS) and NO) (103).

The cytokine-induced anti-parasitic effector mechanisms differ between mice and humans. Even in mice, the functional relevance of anti-parasitic effector molecules is mouse strain-dependent. Whereas, NO is essentially required for intracerebral control of *T. gondii* in susceptible C57BL/6 mice, neutralization of iNOS does not exacerbate chronic TE of resistant BALB/c mice (104). There is good evidence that autophagy-related molecules play a role in parasite killing (105, 106). Furthermore, small GTPases of the IRG family, in particular Irga6 and Irgb6, are crucial for intracellular control of *T. gondii* (107, 108). These IRGs destroy the intracellular replication niche of the parasite by disrupting the PV. Off-target damage of host cell membranes by IRGs is prevented by regulatory GMS-IRGs and mice deficient of regulatory Irgm1 and Irgm3 die from toxoplasmosis due to impaired activity of Irga6 and Irgb6 (109, 110). Parasite-derived rhoptry proteins ROP5 and ROP18 phosphorylate host IRGs such as Irga6 and can thereby inhibit membrane degradation (111). However, this virulence mechanism can be overcome by a highly polymorphic IRG protein (Irgb2-b1) depending on the genetic background of the mouse. Some of its variants can act as decoys for ROP5/18 binding, enabling other IRGs to degrade the PV (112). In addition to IRGs, guanylate-binding proteins (GBP) play an important role in the control of *T. gondii* in mice (113). In part in cooperation with IRGs, GBPs target the PV resulting in killing of the parasite (110). In rats, small GTPAses, NO and also reactive oxygene species contribute to the control of *T. gondii* (114–116).

In contrast to rodents, NO and IRGs play no role for the control of *T. gondii* in humans. In fact, humans are devoid of IRG genes, which are evolutionally maintained only in rodents (117). Thus, the key factors important for the control of *T. gondii* in mice, i.e., TLR11 and TLR12 as well as the IRGs do not exist in humans, which makes it difficult to extrapolate data from the murine model systems of toxoplasmosis to the human disease. In humans, alarmin S100A11 is released from *T. gondii*-infected cells and sensed by monocytes, which upregulated chemokine production to induce recruitment of additional monocytes (118). With respect to human anti-parasitic effector mechanisms, IFN-γ-induced 2,3 indoleamine dioxygenase (IDO) degrades tryptophan and plays a role for parasite control by astrocytes (119). Interestingly, it has been demonstrated that the parasite aims to restrict anti-parasitic IDO levels in the host cell: the *T. gondii* effector molecule TgIST inhibits IDO1 mRNA expression and *T. gondii* GRA15-induced NO antagonizes IFN-γ-dependent IDO levels (120, 121). Importantly, the mechanisms leading to control and elimination of *T. gondii* in murine and human neurons are unknown. Here, a recent study analyzing the transcriptome of *T. gondii*-infected murine neurons, astrocytes, fibroblasts and skeletal muscle cells might provide a source for the identification of host genes important for the control of parasite cysts in neurons (122).

Beside pro-inflammatory cytokines, immunosuppressive cytokines play an important role to prevent immunopathology in toxoplasmosis. The inflammatory monocytes produce IL-10, which prevents an overshooting and immunopathological response in the *T. gondii*-inflamed brain (103). In addition, microglia, macrophages, regulatory B cells, Tbet^+^Foxp3^−^ Th1 cells and some CD8^+^ T cells produce IL-10. IL-10 protects from lethal immunopathology in acute systemic and down-regulates the immune response in chronic cerebral toxoplasmosis (123, 124). In addition, astrocytes produce IL-27, which also inhibits immunopathological Th17 responses in TE (125). In contrast to mice, the CNS immunopathology of human primary toxoplasmosis remains unexplored. In might play a role in some patient categories, e.g., those with epilepsy, since cerebral inflammation is an important trigger of epilepsy and *T. gondii* seropositivity is higher in patients with epilepsy (126–128). As deduced from murine cerebral toxoplasmosis, excessive inflammation might contribute to BBB dysfunction and profound changes in excitatory (in particular glutamate) and inhibitory (in particular GABA) neurotransmission paving the way to epilepsy (129, 130). Interestingly, there is also good evidence that a dysbalance of T cell subpopulations plays a major role in acute and reactivated human OT [reviewed in Maenz et al. (29)].

### Regulation of Cerebral Toxoplasmosis by Brain Parenchymal Cells

In murine TE, strongly activated astrocytes proliferate and form a “ring wall” around *T. gondii*-associated lesions. In the absence of the gp130 receptor, which is important for signaling of cytokines of the IL-6 family, astrocytes become apoptotic resulting in a failure to contain the inflammatory lesions and parasites, with widespread inflammation and finally lethal outcome of TE (131). The important bordering function of astrocytes is further exemplified in mice lacking GFAP, the major intermediary filament of astrocytes. Absence of GFAP in astrocytes results also in increased inflammation and increased parasite loads (132). Additionally, *in vitro* and *in vivo* studies have revealed that astrocytes protect from TE in a STAT1-dependent manner (133) and produce a number of cytokines (IL-1α, IL-6, GM-CSF) and chemokines (CXCL10, CCL2), all with important immunoregulatory functions in TE (134, 135). As mentioned before, astrocytic IL-27 is of key importance to limit activation of the intracerebral immune response. In parallel, suppression of astrocyte activity by TGF-β is required to limit neuroinflammation, to control parasite load and to prevent neuronal damage (125, 136). Thus, astrocytes exert important pro- and anti-inflammatory activities to balance parasite control and intracerebral immune responses. However, although astrocytes can be readily infected *in vitro*, in a mouse infection model neurons rather than astrocytes were targeted by Toxoplasma for infection and injection of effector molecules (26, 137).

In the CNS, neurons are the primary target cell of the parasite. However, the molecular mechanisms of neuronal parasite control remain unclear. Upon *in vitro* infection, neurons produce IL-6, TGF-β_1_, CCL3, and CCL4 (138). Interestingly, expression of the common IL6-cytokine family receptor in neurons is required to prevent hyperinflammation, neuronal loss, parasite replication and, finally, death from TE (139). Thus, the gp130 receptor mediates survival of neurons under inflammatory conditions and is further important for the production of immunosuppressive induction of TGF-β_1_ and IL-27 by neurons.

A third brain parenchymal cell population, strongly activated during TE is the microglia. Microglia are yolk sac-derived cells that develop independently from the bone marrow (140). In murine toxoplasmosis, microglia are strongly activated throughout the entire brain as evidenced by upregulation of MHC class I and II antigens (24, 134). In addition to *de novo* expression of a number of immunological important cell surface molecules, including CD200, CD11a, CD11b, microglia produce several cytokines and chemokines such as IL-6, IL-10, TNF, and GM-CSF in TE and upon infection *in vitro* (141). Functionally important, microglia suppress the proliferation of intracerebral T cells, most probably to prevent excessive T cell proliferation and immunopathology due to the continuous T cell stimulation with the persisting parasite antigens (138).

Data on immune mechanisms leading to the control of intracerebral parasites in humans are scarce. Knowledge is mainly based on extrapolations from reactivated toxoplasmosis in AIDS patients and from severe toxoplasmosis in severely immunosuppressed patients, including HSCT, SOT, immunosuppressive therapy and primary immunodeficiency. Since some cytokines such as IFN-γ, TNF, and IL-12 are key factors for the control *T. gondii* in mice, a high risk for severe primary or reactivated cerebral toxoplasmosis might be expected in patients with primary deficiency for these cytokines and their receptors. However, patients with primary immunodeficiency and severe toxoplasmosis are rarely reported and found in databases. For example, only 2 cases of congenital immunodeficiency–one being primary IFN-γ receptor deficiency- were reported in a multi-center study of 180 cases of toxoplasmosis (142). Furthermore, in a series of 115 patients with IFN-γR1, IFN-γR2, and STAT1-deficiencies, respectively, only one single patient developed clinical toxoplasmosis (143) and among 141 patients with IL-12Rβ1deficiency no single patient developed toxoplasmosis (144). One might also expect that severe toxoplasmosis may manifest upon iatrogenic immunosuppression by T cell-ablating antibodies and anti-cytokine/cytokine receptor therapy. However, although anti-TNF therapy is becoming common, only very few cases of severe toxoplasmosis, reactivated TE or OT have been reported in patients undergoing anti-TNF therapy (145–147). In contrast, mice treated with anti-TNF show an impaired parasite control (148). It has been argued that an underreporting of severe toxoplasmosis in patients treated with biological therapies explains the low number of cases. However, this appears unlikely, since patients under therapy with anti-TNF and other biological therapies are continuously monitored for clinical signs and parameters of infections. In contrast to toxoplasmosis, both humans and mice have an increased risk for tuberculosis under anti-TNF treatment (149), which further highlights the discrepancies between different infections.

What causes this discrepancy between mouse and human data? As excellently delineated in Gazzinelli et al. (117), it is important to keep in mind that rodents play a crucial role in the predator-bait life cycle of the parasite, whereas humans are an accidental intermediate host of *T. gondii*. Therefore, the murine immune system is under continuous evolutionary pressure to develop immunity against newly emerging virulent *T. gondii* strains, which develop in the feline intestine and aim to produce large amounts of parasites in rodents. Consequently, the major murine molecules, i.e., TLR11, TLR12, and IRGs protecting against toxoplasmosis have developed in mice but not in humans.

## Reactivated Toxoplasmosis in the CNS

### Disease and Immunopathogenesis

Groups of patients with severe immunosuppression are at a risk of developing cerebral toxoplasmosis. These include patients with AIDS, SOT, in particular heart transplantation, and HSCT. Additionally, the developing fetus is at risk due to its immature immune system. Because animal models of immunosuppression often use dexamethasone treatment yielding a broad immunosuppression, it is difficult to draw parallels to CD4^+^ T cell deficiency in HIV-infected individuals (21, 22) (Table 1).

In AIDS patients, the reactivation of latent cerebral toxoplasmosis is clinically important. Approximately 30% of *T. gondii* seropositive AIDS patients with a CD4^+^ T cell count below 200/μl develop a reactivated TE, which is lethal if not treated adequately with anti-parasitic drugs (23). Why is the association between reactivated TE and AIDS so strong? The common and most accepted answer is that AIDS patients have a disturbed anti-parasitic T cell response, and, therefore, fail to control the intracellular persisting parasite. Failure to control the parasite in the CNS results in bradyzoite to tachyzoite conversion and a necrotizing TE. However, it has to be kept in mind that *T. gondii* persists in neurons, which can be MHC negative and, therefore, T cells cannot directly interact with neurons in this respect.

Furthermore, AIDS patients harbor a high cerebral viral load due to replication of the virus in microglia and astrocytes (150, 151). Therefore, it is tempting to speculate that (*i*) the virus itself and (*ii*) a dysregulation of microglia and astrocytes may contribute to reactivated TE. In support of this view, in *T. gondii* seropositive patients treated with antibodies depleting T cells or blocking the entry of T cells into the CNS, reactivation of TE does not occur. In fact, only one case of OT has been reported in a multiple sclerosis patient with anti-VLA4 (natalizumab) treatment (152).

One important but rare clinical manifestation is Toxoplasma-associated immune reconstitution inflammatory syndrome (IRIS). Clinically, IRIS can manifest in AIDS patients with reactivated TE and with recent anti-viral therapy. Upon reconstitution of the immune system, T cells may recognize the parasitic antigen and produce large amounts of cytokines, which may cause new neurological symptoms. Additionally, “unmasking” Toxoplasma-associated IRIS may develop in AIDS patients with low CD4^+^ T cell numbers, newly established anti-retroviral therapy and unrecognized TE at the beginning of retroviral treatment. Rarely, AIDS patients with reactivated cerebral toxoplasmosis may develop an infection of the spinal cord (153).

Whereas, reactivated TE ranks among the most common neurological opportunistic infections in AIDS patients, reactivated toxoplasmosis is relatively rare in patients undergoing HSCT and SOT (85, 142). Further, reactivation of latent toxoplasmosis in transplant patients presents preferentially as a systemic infection rather than localized CNS infection (142).

The fetus may become infected if primary infection occurs shortly before or during pregnancy. If the fetus is infected, the parasite has a high predilection for manifesting in the brain and the eyes. Infection early during pregnancy causes more severe symptoms in the fetus and eventually abortion, whereas infections at later time points of gestation cause less severe symptoms (154, 155). Inversely, the risk of infection increases with the time of gestation. The role of fetal immune responses in congenital toxoplasmosis is largely unclear. Most probably, maternal parasite-specific antibodies play a superior protective role.

### Is Chronic CNS Infection “Silent” in Humans?

*T. gondii* has been proposed as a causative agent manipulating the behavior of intermediate rodent hosts to facilitate its parasitic transmission to the definitive feline hosts (71, 156). Also, studies in mice have detected impacts of Toxoplasma infection on dopaminergic, GABAergic and glutamatergic neurotransmission (129, 130, 157, 158). The role of the immune response, and neuronal alterations, in the manipulation of the behavior of rodents during cerebral toxoplasmosis was recently reviewed (5, 7). It should be stressed that data on parasite-mediated modulation of the behavior of rodents cannot be automatically extrapolated to humans, because anti-*T. gondii* immune responses differ fundamentally between mice and men. Humans are accidental intermediate hosts of *T. gondii* while rodent behavior is under evolutionary pressure by the parasite in order to complete the parasitic life cycle. Nevertheless, a recent study in naturally infected chimpanzees also uncovered that *T. gondii* may manipulate the behavior of this primate (159).

Additionally, growing evidence indicates a correlation between *T. gondii* seropositivity and incidence of psychiatric diseases in humans, in particular schizophrenia and bipolar disorders (160–162). Thus, epidemiological studies indicate that carriage of *T. gondii* may increase the risk for some psychiatric diseases and, thus, may serve as co-risk factor. From a clinical perspective, correlative data between psychiatric diseases and toxoplasmosis (or seropositivity) are of importance but need further clinical investigation. Does the combination of *T. gondii* infection together with a specific host genetic profile cumulate in an increased risk of schizophrenia? Does this explain why only some *T. gondii*-seropositive humans suffer from schizophrenia? Are *T. gondii* seropositive patients with schizophrenia a distinct subgroup of patients (even requiring a different treatment regime)? In addition, some studies provide an association between *T. gondii* seropositivity and human behavior or neurocognitive impairment (163–165) while other studies do not find such association (166–168).

Thus, mounting evidences indicate that chronic Toxoplasma infection may not be consistently “silent” in humans, as previously thought. However, at present, compelling evidence for a direct causal link between Toxoplasma infection and mental illness is still missing in humans. It is also not clarified how or to what extent parasite persistence in the CNS may impact on neuropsychiatric disorders or modulate cognitive functions. However, the recently reported intriguing effects on primate behavior might prove useful for further investigations (159). Mechanistic studies are required to elucidate the underlying pathophysiology. Also, prospective studies may elucidate whether the parasite impacts on human behavior or whether behavioral patterns result in a higher risk for *T. gondii* infection.

## Challenges for Applied and Clinical Research

### Therapy

Although treatment failures have been reported for toxoplasmosis, drug resistance does not appear to be a considerable problem in the clinical setting (169). However, a major problem is that tissue cysts are not eliminated by current drug treatments, which only effectively eliminate replicating tachyzoites. The identification of new druggable targets for the bradyzoite cysts would open new pharmacological therapeutic windows (170) and offer the chance to prevent reactivated toxoplasmosis in patients with immunodeficiencies, including HIV infection and organ transplantation, and of recurrent OT in immune-competent individuals. Additionally, *T. gondii* seropositivity has been epidemiologically associated with neurological and mental illnesses, and with cognitive impairment. Presumably, an anti-parasitic therapy eliminating the parasite might be beneficial for these patients and might even answer the question whether *T. gondii* persistence contributes to these disorders.

### Diagnostics

While certain Toxoplasma genotypes have been associated with increased disease frequency and severe manifestations, knowledge on how specific genotypes contribute to the clinical severity is missing in humans. A further understanding of the association between parasite lineages and clinical manifestations, especially severe CNS disease in humans, is therefore needed (171). Further, because the clinical spectrum is contextual, the impact of human genotype on immune-surveillance -especially in relation to mechanisms of parasite reactivation- needs to be explored. Improved diagnostics could be of benefits for risk groups, including monitoring therapy and advice in relation to congenital toxoplasmosis. In addition, the more precise identification of genetic risk factors for reactivated TE in AIDS patients and those undergoing transplantation, might allow a better risk stratification.

### Prevention

Abrogated cyst formation in animals used for meat production would effectively truncate foodborne transmission. Similarly, interruption of the parasitic life cycle by preventing oocyst formation in felines would significantly impact on transmission. To achieve this, a further understanding of the feline parasite stages with a perspective on developing vaccines is needed. Also, an understanding of immune responses in perspective on vaccines directed toward intermediate hosts used for meat consumption.

## Concluding Remarks

It is evident that major advances have been done in the understanding of cerebral toxoplasmosis over the last 3 decades. The use of experimental models has provided extensive new knowledge on mechanisms of disease at the molecular, cellular and systemic levels. Additionally, light has been shed on various aspects of the host-pathogen interplay. Yet, this knowledge needs to be further applied to the understanding of the disease in humans. This still represents a significant challenge for future research.

## Author Contributions

All authors listed have made a substantial, direct and intellectual contribution to the work and approved it for publication.

### Conflict of Interest Statement

The authors declare that the research was conducted in the absence of any commercial or financial relationships that could be construed as a potential conflict of interest.
